# Nurses and an Eight-Hour Day

**Published:** 1920-06-12

**Authors:** M. Mortished

**Affiliations:** Secretary. The Irish Nurses' Union, 29 South Anne Street, Dublin.


*To the Editor of* The Hospital.

### Nurses and an Eight-hour Day.

*To the Editor of* The Hospital.

Sir,—With reference to a recently reported assertion of Sir Lambert Ormsby, to the effect that a nurse while on duty in her ward or in attendance on private patients "half her time on duty might be reading a book, writing a letter, or doing needlework," so long as she were within call of her patients, we venture to state that very few matrons would be satisfied to find a nurse reading a novel in her ward, unless, perhaps, she were on night duty, when such a relaxation might sometimes be considered permissible.

Indeed, few nurses would dare even to sit down in the ward, unless it were to repair or make articles of ward equipment or write a report. Moreover, any time during which a nurse is bound to be on duty, to attend a call or answer a bell, cannot be considered as rest time; and the very fact that she may be called upon to fill every minute, if necessary, with arduous work involving considerable mental strain as well as physical exertion, precludes the possibility of classing a nurse's hours of duty as any but "continuous work."

Emphatically we maintain that it is continuous work, and as continuous work it must be gauged and paid and recognised. Never have the advocates of an eight-hour day for nurses placed them "on a par with the ordinary artisan or builder's labourer," because nurses, in their calling, combine the responsibilities of the manager with the fatigues of the labourer. The nurse, moreover, though nominally on "time work," is, in reality, on "piece work," for her "jobs" *must* be accomplished, at whatever cost to herself. When work is heavy she must work overtime, or at an increased speed, for nothing can be neglected; and for what she cannot find time she must "make time." We feel confident that, in spite of the long silence in which members of the nursing profession have buried their convictions, every nurse can corroborate our view of this question from her own experience.—Yours faithfully,

M. Mortished, Secretary.

The Irish Nurses' Union,
29 South Anne Street, Dublin.
June 7, 1920.

